# Characterization of a novel zebrafish model of *MTMR5*-associated Charcot-Marie-Tooth disease type 4B3

**DOI:** 10.1093/braincomms/fcaf077

**Published:** 2025-02-18

**Authors:** Jordan Lindzon, Maia List, Salma Geissah, Atai Ariaz, Mo Zhao, James J Dowling

**Affiliations:** Program for Genetics and Genome Biology, Hospital for Sick Children, Toronto, ON M5G 0A4, Canada; Department of Molecular Genetics, University of Toronto, Toronto, ON M5S 1A8, Canada; Program for Genetics and Genome Biology, Hospital for Sick Children, Toronto, ON M5G 0A4, Canada; Program for Genetics and Genome Biology, Hospital for Sick Children, Toronto, ON M5G 0A4, Canada; Department of Molecular Genetics, University of Toronto, Toronto, ON M5S 1A8, Canada; Program for Genetics and Genome Biology, Hospital for Sick Children, Toronto, ON M5G 0A4, Canada; Program for Genetics and Genome Biology, Hospital for Sick Children, Toronto, ON M5G 0A4, Canada; Program for Genetics and Genome Biology, Hospital for Sick Children, Toronto, ON M5G 0A4, Canada; Department of Molecular Genetics, University of Toronto, Toronto, ON M5S 1A8, Canada; Zebrafish Genetics & Disease Models Core Facility, Hospital for Sick Children, Toronto, ON M5G 0A4, Canada; Department of Pediatrics, University of Toronto, Toronto, ON M5G 1X8, Canada

**Keywords:** Charcot-Marie-Tooth type 4B3, MTMR5, SBF1, zebrafish

## Abstract

Biallelic loss of expression/function variants in *MTMR5/SBF1* cause the inherited peripheral neuropathy Charcot-Marie-Tooth type 4B3. There is an incomplete understanding of the disease pathomechanism(s) underlying Charcot-Marie-Tooth type 4B3, and despite its severe clinical presentation, currently no disease-modifying therapies. A key barrier to the study of Charcot-Marie-Tooth type 4B3 is the lack of pre-clinical models that recapitulate the clinical and pathologic features of the disease. To address this barrier, we generated a zebrafish Clustered Regularly Interspaced Short Palindromic Repeats/CRISPR-associated protein 9 mutant line with a full gene deletion of *mtmr5.* Resulting homozygous deletion zebrafish are born at normal Mendelian ratios and have preserved motor function. However, starting by 10 days post-fertilization, mutant zebrafish develop obvious morphometric changes in head size and brain volume. These changes are accompanied at the pathological level by abnormal axon outgrowths and by the presence of dysmyelination changes reminiscent of the nerve pathology in human Charcot-Marie-Tooth type 4B3. Importantly, RNA sequencing from brain-enriched samples identifies novel disease pathways including transcriptional changes in genes responsible for neurogenesis, chromatin remodelling/organization, and synaptic membrane homeostasis. Overall, our *mtmr5* knockout zebrafish mirror genetic, clinical and pathologic features of human Charcot-Marie-Tooth type 4B3. As such, it represents a first pre-clinical model to phenocopy the disease, and an ideal tool for future studies on disease pathomechanism(s) and therapy development.

## Teaser

Lindzon *et al*. reported a novel zebrafish *mtmr5/sbf1* knockout model of Charcot-Marie-Tooth type 4B3 that recapitulates key features of the human disorder and provides a first *in vivo* model for therapy development.

## Graphical abstract

**Graphical Abstract:**
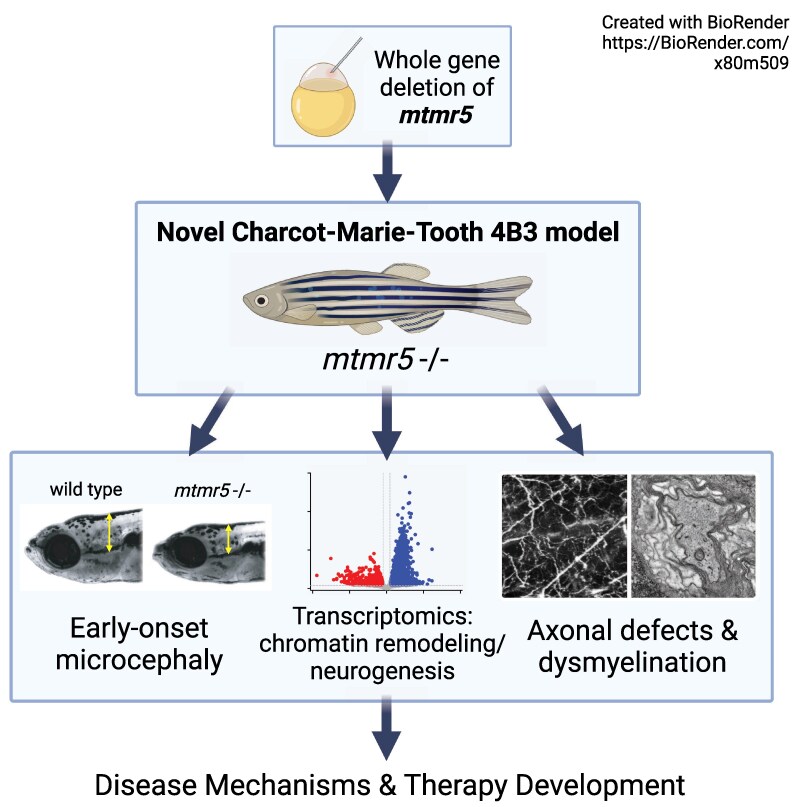

## Introduction

Charcot-Marie-Tooth Disease Type 4B (CMT4B) is a rare, recessive subtype of inherited peripheral neuropathy.^1^ Onset is in infancy or early childhood, and affected individuals experience progressive lower limb weakness and discoordination that ultimately leads to wheelchair dependence. There are a few therapeutic candidates from pre-clinical studies and currently no treatments for CMT4B.

There are three genetic subtypes of CMT4B: CMT4B1 (due to biallelic mutations in *MTMR2*),^2^ CMT4B2 (due to biallelic mutations in *MTMR13/SBF2*)^3^ and CMT4B3 (due to biallelic mutations in *MTMR5/SBF1*).^4^ All three subtypes share elements of peripheral nerve pathology (including characteristic myelin outfoldings), and all three are assumed to have common pathomechanisms, as the causative genes encode members of the myotubularin-related family of phosphoinositide phosphatases (MTMRs). Phosphatase active MTMRs (such as MTMR2) dephosphorylate PI3P and PI(3,5)P2, and are involved in the regulation of vesicle trafficking through the endolysosomal compartment.^5^ Phosphatase inactive MTMRs are primarily thought to regulate the localization and/or activity of phosphatase active MTMRs. Both MTMR5 and MTMR13 directly interact with MTMR2 through their coiled-coil domains to form heterodimers, and both are proposed to function as regulators of MTMR2 activity.^6,7^

CMT4B3 is unique as compared with CMT4B1/2 because patients experience signs and symptoms unrelated to peripheral demyelination. These unique clinical features include axonal peripheral neuropathy,^8^ structural brain changes (fork and bracket syndrome),^9^ intellectual disability^10^ and alterations in skeletal muscle. The expanded phenotype of CMT4B3 suggests that MTMR5 has functions beyond those specifically related to MTMR2 and also that therapies targeted at CMT4B1/2 may not fully address critical aspects of the CMT4B3 clinical syndrome.

To date, no pre-clinical models of CMT4B3 recapitulate the key features of the human disease. *Mtmr5* knockout mice have been generated; they have defective spermatogenesis,^11,12^ and peripheral axon demyelination without overt axon defects or brain involvement.^12^ Zebrafish serve as an excellent alternative model organism for studying CMT4B3. They offer the advantage of rapid development, large numbers of offspring, easily detected and quantifiable motor phenotypes and facile genetic manipulation.^13^ Zebrafish have previously been shown to accurately model other subtypes of CMT, particularly ones with axonal involvement (such as CMT2A),^14^ and to be useful for studying the function of MTMRs^15^ and myelination.^16^ More importantly, zebrafish are an ideal organism for *in vivo*, whole animal drug screening^17^ and are unique among genetically tractable vertebrate model systems for this ability.

**Figure 1:**
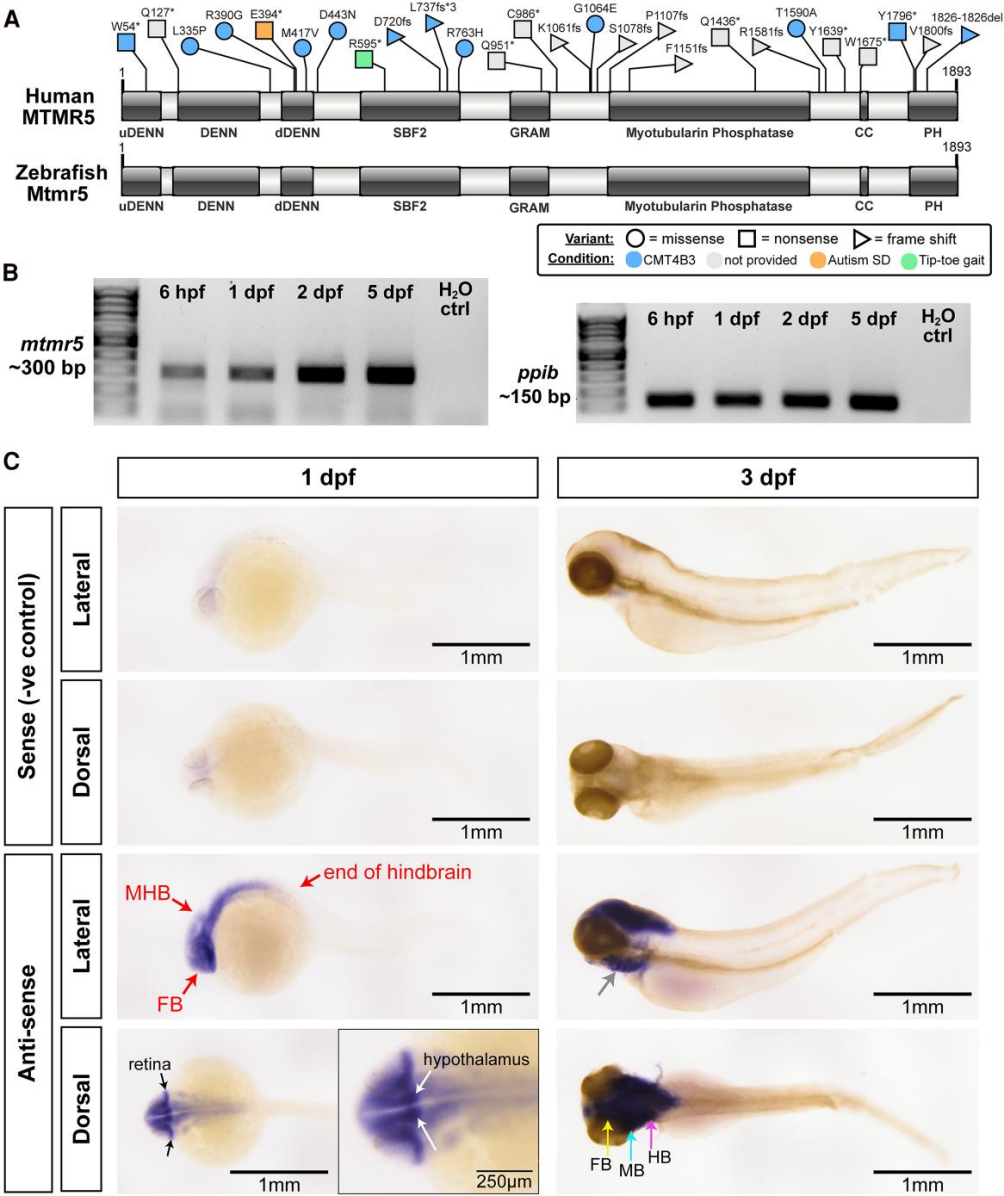
**MTMR5 domain topology, pathogenic variants, and spatiotemporal expression pattern in zebrafish.** (**A**) Human MTMR5 contains six functional domains: an N-terminal tripartite DENN domain [upstream DENN (a.a. 1–86), DENN (a.a. 129–311) and downstream DENN (a.a. 364–433)], an SBF2 domain (a.a. 543–765), a GRAM domain (a.a. 883–969), a catalytically inactive myotubularin-phosphatase domain (a.a. 1108–1533), a coiled-coil domain (CC, a.a. 1651–1665) and a C-terminal PH domain (a.a. 1763–1868). Of note, the domain topology of the MTMR5 protein is highly conserved between human and zebrafish. Pathogenic variants are found throughout the gene (for details, see Supplementary Table 1). Note: autism SD (spectrum disorders). (**B**) RT-PCR using total cDNA shows temporal expression pattern of zebrafish *mtmr5* from 6 h post-fertilization (hpf) to 5 dpf (days post-fertilization). Note: *ppib* is a housekeeping control. (**C**) Whole-mount *in situ* hybridization using DIG-conjugated RNA probes in 1 dpf and 3 dpf embryos. The sense probe (negative or −ve control) gives no staining at 1 dpf or 3 dpf. At 1 dpf, from the lateral view, the anti-sense probe shows ubiquitous staining in the brain, including the forebrain (FB), midbrain-hindbrain boundary (MHB), and the hindbrain; the dorsal view at 1 dpf also revealed staining in fine structures such as the retina and the hypothalamus (inset scale bar 250 μm). Similar brain-specific staining can be observed at 3 dpf for the anti-sense probe, in the forebrain (FB), midbrain (MB) and hindbrain (HB). Staining is also visible in the jaw region at 3 dpf (lateral view, arrow). Scale bars: 1 mm. Domain topology of MTMR5 proteins was mapped using SMART^18^ and DeepCoil.^19^ Domain illustrations were generated using IBS 2.0.^20^ Uncropped gels of Fig. 1B are included in Supplementary Fig. 5A.

Similar to humans, zebrafish possesses one copy of the MTMR5 gene. MTMR5 proteins are highly conserved from zebrafish (NP_001038623.1) to human (NP_001352748.1) (72.13% identity), and both contain six functional domains (Fig. 1A): a tripartite DENN (Differentially Expressed in Neoplastic versus Normal) domain that regulates membrane trafficking by mediating Rab GTPase activity,^21,22^ a SBF2 (SET binding factor) domain, two phosphoinositide/membrane-binding domains consisting of a GRAM domain and a pleckstrin homology (PH) domain, a pseudo/inactive myotubularin phosphatase domain, and a coiled-coil (CC) domain that forms heterodimers with MTMR2.^6^ Of note, the presence of an N-terminal DENN domain is a unique feature of MTMR5 and MTMR13 in the myotubularin family.^23^ Whilst missense CMT4B3 patient variants^4,8-10,24-27^ are clustered in the DENN domains and the SBF2 domain (Fig. 1A), suggesting their importance in normal function of MTMR5, pathogenic variants are reported throughout the gene^28^ and are generally associated with loss of expression and/or function (see Supplementary Table 1 for detailed information on the pathogenic variants).

In this study, we generated and characterized a novel zebrafish model of MTMR5-associated CMT4B3. We established a zebrafish *mtmr5* knockout (*mtmr5*-KO) line via CRISPR (Clustered Regularly Interspaced Short Palindromic Repeats)/Cas9 (CRISPR-associated protein 9)-mediated full gene deletion and confirmed the absence of *mtmr5* mRNA transcripts in the resulting homozygous mutants. Phenotypic characterization revealed that *mtmr5*-KO zebrafish recapitulate many key features of CMT4B3, including microcephaly, axonal defects and dysmyelination. Additionally, brain-enriched RNA sequencing identified novel dysregulated pathways in the *mtmr5*-KOs, including chromatin remodelling/organization and synaptic biogenesis. In total, our findings establish *mtmr5*-KO zebrafish as an ideal model for studying MTMR5-associated CMT4B3 pathomechanisms and for identifying potential therapies.

## Materials and methods

### Zebrafish maintenance and collection

Zebrafish (AB strain) were raised and maintained at 28.5°C at the Zebrafish Facility at the Hospital for Sick Children, Toronto, ON, Canada. Experiments were performed on zebrafish embryos and larvae (grown in blue water, 0.3 g/L Instant Ocean, 1 mg/L methylene blue, pH 7.0) from the one-cell stage up to 14 dpf. All zebrafish procedures were performed in strict accordance with the Animals for Research Act of Ontario and the Guidelines of the Canadian Council on Animal Care.

### RNA extraction, cDNA synthesis and PCR

Total mRNA was isolated from staged zebrafish larvae (*n* = 10–25 per sample) using an RNeasy Mini Kit (Qiagen, 74106) and reverse-transcribed with iScript (Bio Rad, 1708891). PCR was performed using GoTaq Green Master mix (Fisher Scientific, PR-M7123) and a PCR cycler (Applied Biosystems). For studying the temporal expression pattern of *mtmr5*, primers used are as follows: *mtmr5* forward 5′-AAGCATCAGAACATCTGCCG-3′, reverse 5′-TGTTTTTGGCAGAGACAAGAAGT-3, amplifying a 305 bp product targeting exon 42 (Ensembl transcript ID: ENSDART00000159762.2). For comparing *mtmr5* transcript levels in WT versus *mtmr5-*KOs, primers used are as follows: forward 5′-TGAGCACCTCAGGGAAATGG-3′, reverse 5′- AAAGAGTCACCGCAGGCTAA-3′, amplifying a 106 bp product targeting exons 28/29 (Ensembl transcript ID: ENSDART00000109452.5). The housekeeping control is *ppib,* and the primers used are as follows: forward 5′-ACCCAAAGTCACGGCTAAGG-3′, reverse 5′-CTGTGGTTTTAGGCACGGTC-3′. Protocol conditions for PCR were the following: denaturation at 95°C for 5 min; annealing at 58°C for 30 s, extension at 72°C for 45 s, followed by 35–40 cycles; and final extension at 72°C for 5 min.

### Whole mount *in situ* hybridization

DNA template was generated via pGEM T-easy cloning system (Promega, A1360), where the insert was generated by PCR from 2 dpf total cDNA synthesized using iScript (Invitrogen, 11755050). Primers were designed to target a ∼1 kb region at the 3′UTR of zebrafish *mtmr5* mRNA transcript: forward 5′-CCTCATAGCCAATGGGGAGC-3′, and reverse 5′-GCTGGATATCGGAAGCGGAT-3′. Digoxigenin (DIG)-labeled *in situ* probes were synthesized using DIG RNA Labeling Kits (Roche, 11277073910). RNA *in situ* hybridization was carried out as previously described.^29^ Briefly, 1 dpf and 3 dpf AB embryos were fixed in 4% paraformaldehyde (PFA) for 2 h at room temperature and then dehydrated in 100% methanol at −20°C until needed. Embryos were then permeabilized using Proteinase K (Thermo Scientific, EO0491) and incubated with DIG-labeled antisense RNA probes in hybridization solution. Hybridizations of the probe with the RNA were detected with an alkaline phosphatase-conjugated antibody (1:5000; anti-DIG-AP, Fab Fragments, Roche, 11093274910). Finally, stained embryos were cleared in a gradient PBST (0.1% Tween 20 in PBS)/100% methanol wash, stored in BBA (2:1 Benzyl benzoate:Benzyl alcohol) overnight and imaged in BBA the next day under a ZEISS AxioZoom stereomicroscope.

### CRISPR/Cas9 genome editing and full gene deletion

The program Chopchop (http://chopchop.cbu.uib.no/)^30^ was used to design each of the guide RNAs (gRNAs) used in this project. Next, 50–100 one-cell-stage WT embryos were injected with the gRNA (150 pg per embryo) and Cas9 mRNA (100 pg per embryo) with a Picopump (World Precision Instruments). Genomic DNA was extracted using 50 L of NaOH solution (50 mM) at 95°C for 20 min, and incubated at 4°C for 10 min, followed by neutralization using 50 µL of Tris-HCl (1 M, pH 8.0). gRNA efficiency was determined using HRM analysis performed on a Roche Lightcycler 96. Once highly efficient gRNAs was identified at the desired genomic region, potential founders (F0) were outcrossed to WT AB zebrafish. In-cross progeny from the F3 and F4 generations were used for the characterization of the *mtmr5* mutant phenotype. To create full gene deletion mutants, the gRNAs used in this study were: gRNA (left) 5′-CGTTGTAGTCGGCTACGATC-3′ at exon 1, and gRNA (right) 5′-CCTATCTGATGCGTAGGTGT-3′ at exon 42 (last exon). Primers used for determining cutting efficiency via HRM were as follows: gRNA (left) forward 5′-TGGAAAGACAGTCATCCCCG-3′, reverse 5′-GCTGGCTGTGTCTCACTCTTA-3′; gRNA (right) forward 5′- CTCAAGACGACCAAAAGAGTGT-3′, reverse 5′- GCGTCACAGTTATGTTGTCTGC −3′. Primers used for determining full-gene deletion (i.e. genotyping) via PCR were as follows: common forward 5′-TATCTTCGTGCACGCGCTGTAA-3′, wild-type-specific reverse 5′-GCTGGCTGTGTCTCACTCTTA-3′ (328 base-pair product when paired with common forward); deletion-mutant-specific reverse 5′- GAAGGAAGTGTTTTGAAGCCGA-3′ (499 base-pair product when paired with common forward). Genotyping was performed on extracted genomic DNA as previously described.^31^

### Gross morphology measurements

Genotyped zebrafish from *mtmr5*-heterozygous in-crosses were treated with tricaine (1:25 in embryo water) and live-imaged in 1% low-melt agarose under AxioZoom stereoscope (ZEISS). Brightfield images were then analyzed using Line Tool in Fiji ImageJ to measure brain length, brain height and body length. All measurements were normalized to the average of the wild-type sibling and entered into Prism 9 (GraphPad). One-way ANOVA analysis was performed to analyze the statistical significance between wild-type, heterozygous and knockout conditions.

### Swim assay

To quantify muscle performance, 3–14 dpf zebrafish were individually transferred to a 96-well plate. Three days post-fertilization zebrafish were incubated in an optovin analogue 6b8 (10 μM in 200 μl embryo water, ChemBridge, 5707191) at 28.5°C for 5 min in the dark. Motor activity of the larvae was recorded and analyzed using ZebraBox (Viewpoint, France) as previously described^32^ with 30 s light on, 1 min light off, 30 s light on, 1 min light off and 30 s light on. Without optovin incubation, 6 dpf or 14 dpf zebrafish were tracked in the 96-well plate in the ZebraBox (dark) for free swim for 2 h at room temperature. Three independent experiments were conducted. Total distance travelled (mm) was plotted and analyzed using Prism 9 (GraphPad). For each group, mean±SEM was calculated, and one-way ANOVA was performed to test statistical significance.

### Apoptosis assay

To visualize apoptotic cells, live zebrafish (2 dpf, 7 dpf or 14 dpf) were incubated with acridine orange (5 μg/mL; Catalog no. A1301, Invitrogen) for 30 min at 28.5°C (in dark) and washed 3 × 5 min with embryo water before being embedded in 1% low melting agarose (containing 1:25 tricaine) for confocal live imaging (Leica SP8, 10 × air objective, NA = 0.4). Average *Z*-projections were generated, and gray values (intensity) of forebrain, midbrain and hindbrain were measured using Fiji ImageJ. Data were entered into Prism 9 (GraphPad). Student’s *t*-test was performed to examine statistical significance.

### Whole mount immunofluorescence

Seven days post-fertilization zebrafish were genotyped and fixed with 4% PFA for 2 h at room temperature (for phalloidin and myosin A4.1025), or 100% methanol (MeOH, for acetylated alpha tubulin) for 20 min to 2 h at room temperature followed by storage at −20°C for at least overnight. Methanol fixed samples were gradually brought back to PBSTween (0.1%) using gradient PBST/MeOH wash followed by PBST wash 3 × 5 min. Samples were then permeabilized by 5 min H_2_O wash at room temperature, 1 h 100% acetone incubation at −80°C and then 5 min H_2_O wash at room temperature. After a brief PBST wash, samples were then blocked (2% goat serum, 1% BSA, 1% DMSO in 1× PBS buffer) for at least 2 h at room temperature. Samples were incubated with primary antibodies: mouse anti-myosin (1:10, A4.1025, DSHB), mouse anti-synaptotagmin 2 (1:10, Znp-1, DSHB), or mouse anti-acetyl-tubulin (1:200, T7451, Sigma) overnight at 4°C. After 4 × 15 min PBST wash, embryos were then incubated with Goat anti-Mouse IgG (H + L) Alexa Fluor 488 (1:150, A28175, Invitrogen), alpha-bungarotoxin (1:150, Alexa Fluor 555, B35451, Invitrogen) or Alexa Fluor 555 Phalloidin (1:500, A34055, Invitrogen) overnight at 4°C. To examine brain ventricle size, genotyped and PFA fixed 7 dpf zebrafish were directly incubated with DAPI (10 μM in blocking solution) overnight at 4°C. After 4 × 15 min PBST wash, embryos were embedded in 1% low melting agarose and imaged using Leica SP8 confocal microscope (40 × water immersion, NA = 1.10; 1024 × 1024; step size 0.42 μm). Standard deviation *Z*-projections were generated and analyzed by Threshold (default) → Binary → Skeletonize Plugin → Analyze Skeleton in Fiji Image J. The total (sum) number of branches, the total number of end-points and the average of branch length were then extracted from Fiji ImageJ and analyzed using Excel, followed by statistical significance calculation using Student’s *t*-test in Prism 9 (GraphPad).

### Transmission electron microscopy

The transmission electron microscopy experiment was performed as previously described.^32^ Briefly, genotyped 14 dpf larvae were anaesthetized using 0.1% tricaine and fixed in Karnovsky’s fixative at room temperature for 2 h and re-fixed in fresh fixatives overnight at 4°C. Samples were post-fixed with 1% osmium, dehydrated with serial ethanol wash and infiltrated with Epon. Ultrathin sections (60 nm) were cut using Leica Ultracut ultramicrotomes. Grids were post-stained and imaged using a Hitachi HT7800 transmission electron microscope.

### RNA sequencing and analysis

#### Sample preparation

Zebrafish larvae were fin clipped and genotyped at 4–6 dpf. At 7 dpf, *n* = 11–17 zebrafish heads were dissected per genotype and immediately frozen at −80°C. Four biological replicates were included from different breeding pairs over time. RNA extraction was performed for all samples (4 × WT, and 4 × *mtmr5*-KO) using the RNeasy kit (Qiagen). RNA samples were sent to The Centre for Applied Genomics—Next-Generation Sequencing Facility (PGCRL, The Hospital for Sick Children, Toronto) for paired-end sequencing using Illumina NovaSeq 6000 platform. Quality control, RNA library preparation, cDNA synthesis, and library validation were performed as previously described.^33^

#### RNA sequencing library and reference genome information

Type of library: paired end. Genome reference sequence and annotations: GRCz11/Ensembl release 113 (primary assembly), downloaded on 21 October 2024.

#### Read pre-processing, alignment and obtaining gene counts

The sequencing data are in FASTQ format. Read pre-processing, alignment, and gene count enumeration were performed using Galaxy (https://galaxyproject.org).^34^ The quality of the data was assessed using FastQC, and adaptors were trimmed using Trim Galore. The raw trimmed reads were aligned to the reference genome using RNA STAR. The filtered STAR alignments were processed to extract raw read counts for genes using htseq-count. Assigning reads to genes by htseq-count was done in the mode ‘intersection_nonempty’ and only uniquely mapping reads were counted.

#### Exploratory analysis and differential gene expression analysis

Exploratory analysis was performed using R v4.4.0. Reference- and count-based differential gene expression analysis was performed using DESeq2.^35^ For each gene, a minimum expression cut-off of 10 read counts in at least 3 samples was used. Gene ontology analyses were performed using cluserProfiler.^36^ Adjusted *P*-values were calculated using Benjamini & Hochberg method.

### Protein extraction

Total zebrafish proteins were extracted as previously described^31^ using 7 dpf WT or *mtmr5-*KO larvae. Briefly, genotyped 7 dpf WT or *mtmr5* larvae were collected (*n*∼20 per group) and immediately stored at −80°C. Samples were homogenized in 1 × RIPA buffer (Cell Signaling Technology, Catalog no. 9806) supplemented with Complete Mini EDTA-free Protease Inhibitor tablets (Roche, 11836170001) and phosphatase inhibitors (Sigma-Aldrich, 524625, 1:100) and then centrifuged at 4°C for 10 min. Supernatants were collected, and protein concentration quantified using a Pierce^TM^ BCA protein assay kit (Thermo Fisher Scientific, 23225).

### Western blotting

Western blotting was performed as previously described.^31^ Briefly, protein lysates (40 μg/lane) were boiled at 95°C for 5 min before loading. Samples were run at 100 V, transferred using semi-dry method at 10 V for 70 min and resolved on PVDF membranes. Total protein staining was performed using REVERT^TM^ 700 (Li-Cor) prior to blocking. Membranes were blocked in 1 × TBST containing 3% BSA for 2 h at room temperature and then incubated overnight at 4°C with primary antibody. Membranes were washed and probed with secondary antibody for 1 h at room temperature. After incubating with Clarity Max^TM^ ECL (BioRad), membranes were imaged using the Gel Doc^TM^ System (BioRad). Primary antibody used: anti-LC3B (1:1000; Abcam, ab51520). Secondary antibody used: anti-rabbit-HRP (1:5000; 1706515, Bio-Rad).

## Results

### *Mtmr5* is predominantly expressed in the zebrafish brain

To examine the spatiotemporal expression pattern of *mtmr5* in zebrafish, we performed RT-PCR and detected *mtmr5* mRNA from 6 h post-fertilization (hpf; Fig. 1B) through to 5 days post-fertilization (dpf). We also performed whole mount *in situ* hybridization on 1 dpf and 3 dpf zebrafish embryos using an antisense DIG-labelled RNA probe designed against the 3′UTR of *mtmr5* transcript (with a sense probe to the same sequence as a negative control). Whilst the sense control showed no staining, the anti-sense probe showed strong staining, predominantly in the brain, at both 1 dpf and 3 dpf (Fig. 1C). The staining was not restricted to a brain region but instead was ubiquitously present in the forebrain, the midbrain, and the hindbrain.

### Generation of a zebrafish *mtmr5* knockout model via CRISPR/Cas9 genome editing

**Figure 2:**
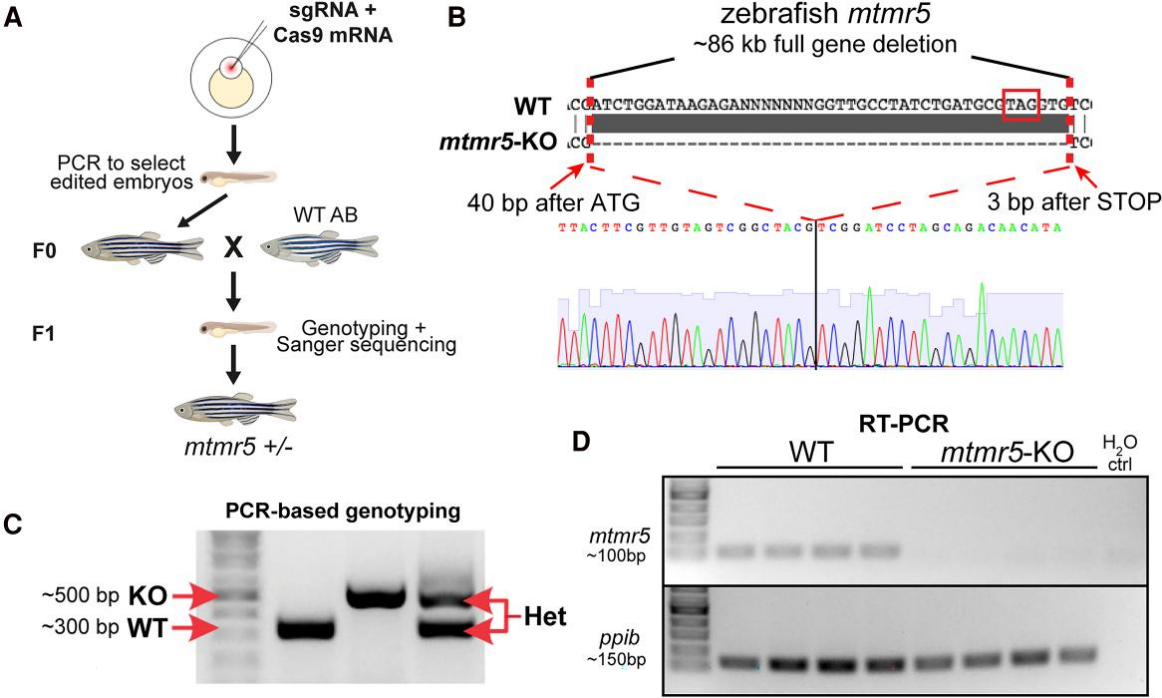
**Generation of a full *mtmr5* gene deletion/knockout zebrafish.** (**A**) Schematic diagram of the workflow. Single-guide RNAs (sgRNA) and Cas9 (Clustered regularly interspaced short palindromic repeats or CRISPR associated protein 9) mRNA were co-injected into 1-cell stage zebrafish embryos (wild-type or WT; AB strain). The full *mtmr5* gene deletion was first identified by PCR at F0 and further confirmed at F1 by PCR and Sanger sequencing. (**B**) Sanger sequencing revealed an 86 kilobase (kb) deletion at the *mtmr5* gene, starting 40 base pairs (bp) after the translation start site to 3 bp after the translational stop site (STOP). (**C**) Genotyping was achieved using a 3-primer PCR method, showing the WT band at 300 bp (one flanking and one inside *mtmr5*), the mutant band at 500 bp (primers flanking *mtmr5*) and the heterozygous (Het) bands at both 300 and 500 bp. (**D**) Reverse transcription (RT)-PCR using specific primers against the deleted *mtmr5* region shows a ∼100 bp band in the 7 dpf (days post-fertilization) WT cDNAs, and the lack of amplification in the mutant cDNAs. Housekeeping control (*ppib*) shows bands in both WT (wild-type) and *mtmr5*-knockout (KO) samples, and H_2_O (water) control shows no bands. Note: four biological replicates per condition, each lane represents *n* = 15–20 zebrafish heads (7 dpf). Uncropped gels of Fig. 2C and D are included in Supplementary Fig. 5B and C, respectively.

In order to study MTMR5 function, we generated a zebrafish line with a full gene deletion of *mtmr5.* A ∼86 kb region (encompassing 40 bp before the translation start site through 3 bp after the stop codon) was removed from the genome using Clustered Regularly Interspaced Short Palindromic Repeats/Cas9 genome editing. Briefly, we designed and tested two highly efficient single guide RNAs (sgRNAs) against the zebrafish *mtmr5* gene, one cutting near the translation start codon (ATG), and the other after the stop codon (TAG). sgRNAs were co-injected with Cas9 mRNA into 1-cell stage wild-type embryos, and injected embryos were raised to adulthood (Fig. 2A). Complete deletion of the *mtmr5* genomic region was confirmed by Sanger sequencing (Fig. 2B). PCR using primers flanking the *mtmr5* gene were used to screen adult founders (F0) for carrying a ∼500 base pair (bp) band and genotyping subsequent generations (Fig. 2C). F0 founders were outcrossed with wild-type fish to establish germline transmission of the deletion, and subsequent F1 heterozygous fish were used to establish homozygous deletion mutants. All subsequent studies were done on F3 generation and beyond. To confirm the absence of *mtmr5* transcript, RT(reverse transcription)-PCR was performed on RNA isolated from wild-type and mutant (homozygous for the deletion) zebrafish at 7 dpf, and primers binding to exon 28/29 within the wild-type *mtmr5* transcript were used. Whilst housekeeping control was detected in all samples, no *mtmr5* amplification was detected in the mutant, an anticipated consequence for a full gene deletion (Fig. 2D). We thus successfully generated an *mtmr5* knockout (KO) zebrafish line via CRISPR/Cas9-mediated full gene deletion.

### Loss of *mtmr5* is associated with early-onset microcephaly and an overall reduction in size, but no changes in survival or motor function

*mtmr5*-KO zebrafish are viable, can survive to adulthood, and can successfully breed. To see if loss of *mtmr5* impacts zebrafish survival, we tracked a cohort (*n* = 56) of fish over 2 weeks. Between 5 dpf and 14 dpf, the number of KOs declined from 56 to 49, a ∼13% loss of fish that is more than what would be anticipated in wild-type (WT) fish (0–2%). From 14 dpf onward, there is no clear difference in survival, with the oldest recorded *mtmr5*-KO fish being 1.5 years (the maximum age allowed in the zebrafish facility). We also observed no overt abnormalities in their swim behaviours at 4 months (Supplementary Video 1) and at 1.5 years (Supplementary Video 2) but did note that *mtmr5*-KO fish tend to be found closer to the water surface, perhaps related to challenges acquiring food.

**Figure 3:**
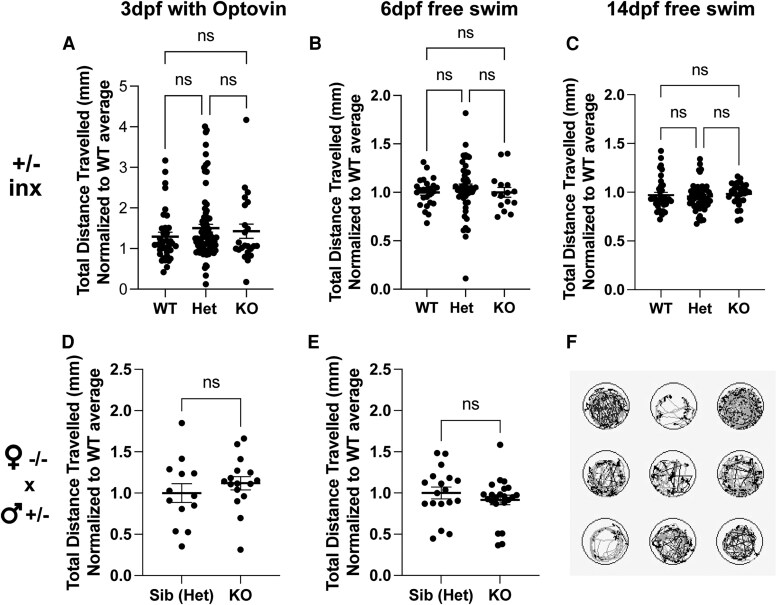
**Loss of *mtmr5* does not affect motor functions.** (**A**) Embryos generated from a *mtmr5*-heterozygous (+/−) in-cross (inx) were subjected to Optovin treatment at 3 days post-fertilization (dpf) followed by the quantification of the total distance travelled (mm) by each embryo via ZebraBox. There was no apparent difference in swim performance between wild-type (WT) *n* = 36, heterozygous (Het) *n* = 77, and knockouts (KO) *n* = 23. (**B** and **C**) Embryos resulting from a heterozygous in-cross were subjected to a free swim assay (no Optovin treatment) at (**B**) 6 dpf (WT *n* = 25, Het *n* = 47, KO *n* = 15) and (**C**) 14 dpf (WT *n* = 35, Het *n* = 30, KO *n* = 15). There were no observed differences in swim performance between the three genotypes at both developmental stages. **(D** and **E**) Embryos resulting from a homozygous knockout female crossed with a heterozygous male were subjected to swim assays at (**D**) 3 dpf (with Optovin treatment; Sib (Het) *n* = 13, KO *n* = 16), and at (**E**) 6 dpf (free swimming assay) (Sib (Het) *n* = 17, KO *n* = 22), and again, at both developmental time points, there were no differences in swimming performance between the genotypes. (**F**) A representative image of the swim trace tracking used by the ZebraBox program to quantify the total distance travelled (mm) for each embryo per well. All statistical analyses include at least three independent experiments. Each dot on the graphs represents one zebrafish, at least *n* = 4 zebrafish were used per group per experiment. Quantitative data are mean±SEM, normalized to the average of WT and presented as ratios. One-way ANOVA analysis was performed for all heterozygous in-cross experiments and an unpaired two-tailed Student’s *t*-test was used for the homozygous female × heterozygous male experiment; ns = not significant, *P* > 0.05.

To dissect *mtmr5* loss-of-function effects on neuromuscular function, we performed swim assays on 3 dpf, 6 dpf, and 14 dpf zebrafish using ZebraBox (Viewpoint) (Fig. 3A–F). ZebraBox is a semi-automatic platform that can be used to track the movement of individual embryo/larva in 96-well plates. For 3 dpf zebrafish embryos (which have limited spontaneous activity at baseline), we induced movement using optovin,^32^ while in 6 dpf and 14 dpf zebrafish, we tracked spontaneous swimming. We compared total distance travelled and average speed in WT siblings versus *mtmr5*-KOs generated from heterozygous in-crosses and observed no difference in motor performance (total distance in Fig. 3A–C, average speed in Supplementary Fig. 1A). We further compared the distance travelled at small (<5 mm/s), medium (>5 mm/s, < 20 mm/sec) and large (>20 mm/s) speeds at 3 dpf and detected no significant differences (Supplementary Fig. 1B–D). In order to remove the contribution of maternally deposited *mtmr5*, we further characterized offspring from homozygous females crossed to heterozygous males, and again observed no difference in swim performance (Fig. 3D and E). Moreover, we performed whole mount immunofluorescence staining on 7 dpf zebrafish to examine sarcomeric integrity/muscle mass (phalloidin), and observed no defects in the KO (Supplementary Fig. 2). In all, similar to observations from *Mtmr5*−/− mice,^12^ the loss of *mtmr5* does not cause significant sarcomeric or movement defects in the zebrafish larvae.

**Figure 4:**
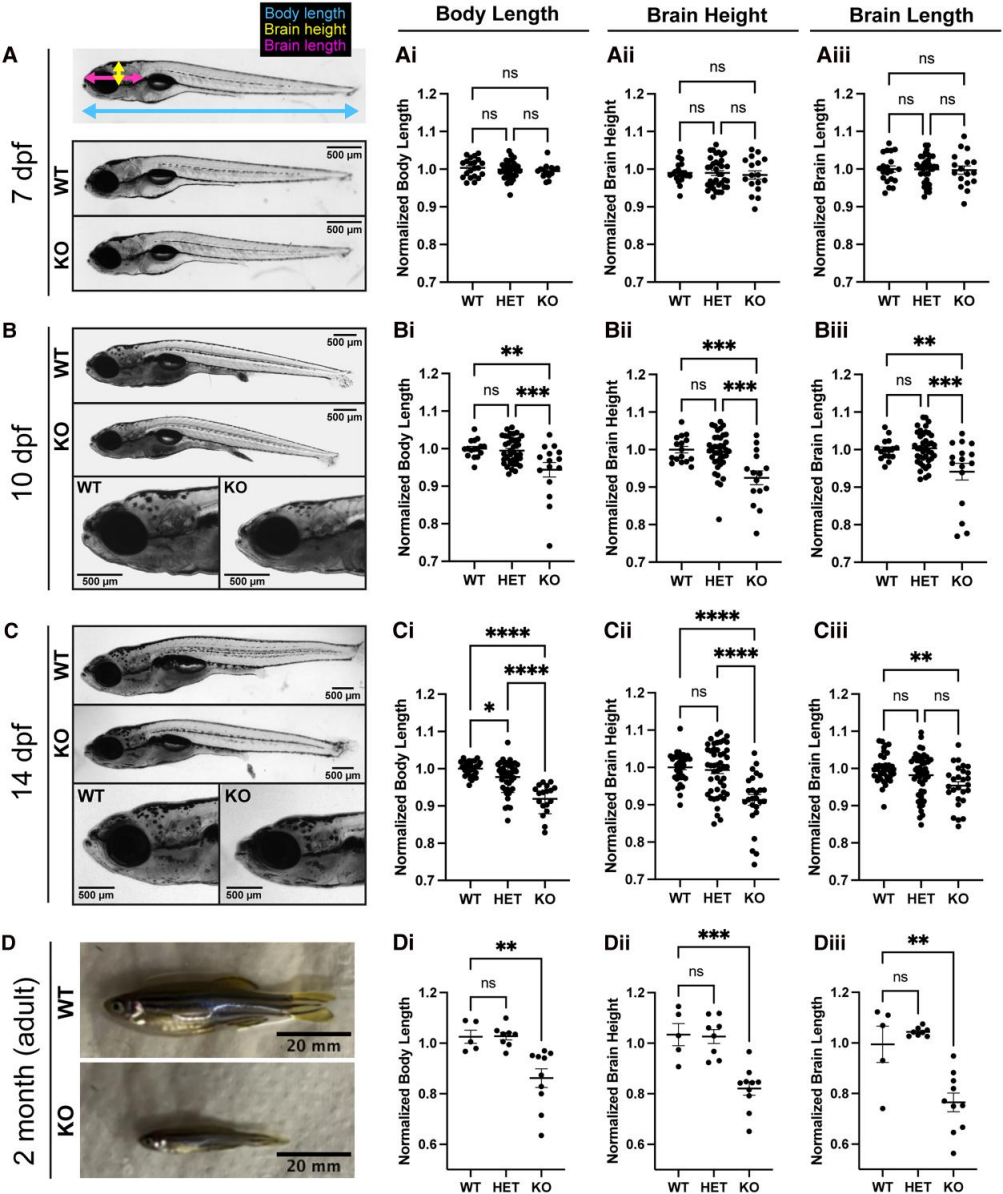
**Loss of *mtmr5* results in microcephaly and an overall body size reduction from as early as 10 dpf.** (**A**, top) A representative image of a WT 7 dpf zebrafish with lines delineating the measured gross anatomy. This system is used to measure all age groups (7 dpf, 10 dpf, 14 dpf and 2 months). (**A**, middle and bottom) At 7 dpf, there was no observed difference in (**Ai**) body length, (**Aii)** brain height or (**Aiii**) brain length between the siblings and KO. WT *n* = 20, heterozygous (Het) = 36, and KO *n* = 17. (**B**) Microcephaly (i.e. reduced absolute brain size) starts to appear in the KO at 10 dpf, when **(Bi)** there was a significant reduction in body length, **(Bii)** brain height and **(Biii)** brain length in the KO. WT *n* = 16, Het *n* = 40, KO *n* = 16. (**C** and **D**) Similarly, at 14 dpf (**C**) and at 2 months (**D**), there was a significant decrease in body length (**Ci** and **Di**), brain height (**Cii** and **Dii**) and brain length (**Ciii** and **Diii**) in KO compared to WT siblings. 14 dpf: WT *n* = 27, Het *n* = 45, KO *n* = 18. 2 months: WT *n* = 5, Het *n* = 8, KO *n* = 10. All statistical analyses include at least three independent experiments. Each dot on the graphs represents one zebrafish, at least *n* = 5 zebrafish were used per group per experiment. Quantitative data are mean±SEM, normalized to the average of WT and presented as ratios. One-way ANOVA was done for all measurements. *P < 0.05; **P < 0.01; ****P < 0.0001; ns, not significant. Scale bars: 500 μm (7, 10 and 14 dpf images) or 20 mm (adult images).

We then characterized the gross morphology of *mtmr5*-KO zebrafish by measuring brain and body size at different developmental stages. At 7 dpf, no changes in brain or body size were observed in *mtmr5*-KO zebrafish compared to WT and heterozygous (Het) siblings (Fig. 4A). Starting at 10 dpf, we noted a significant reduction (∼10%) in the body length of *mtmr5*-KO compared with siblings (Fig. 4Bi), as well as a significant reduction (∼10%) in brain height (Fig. 4Bii) and brain length (Fig. 4Biii), indicating the presence of a small brain phenotype (i.e. microcephaly). These features were also present in 14 dpf zebrafish and beyond into adulthood (Fig. 4C and D). The reduced overall body size of the *mtmr5-*KO zebrafish is consistent with the overall smaller body size reported in *Mtmr5* −/− adult mice.^12^

**Figure 5:**
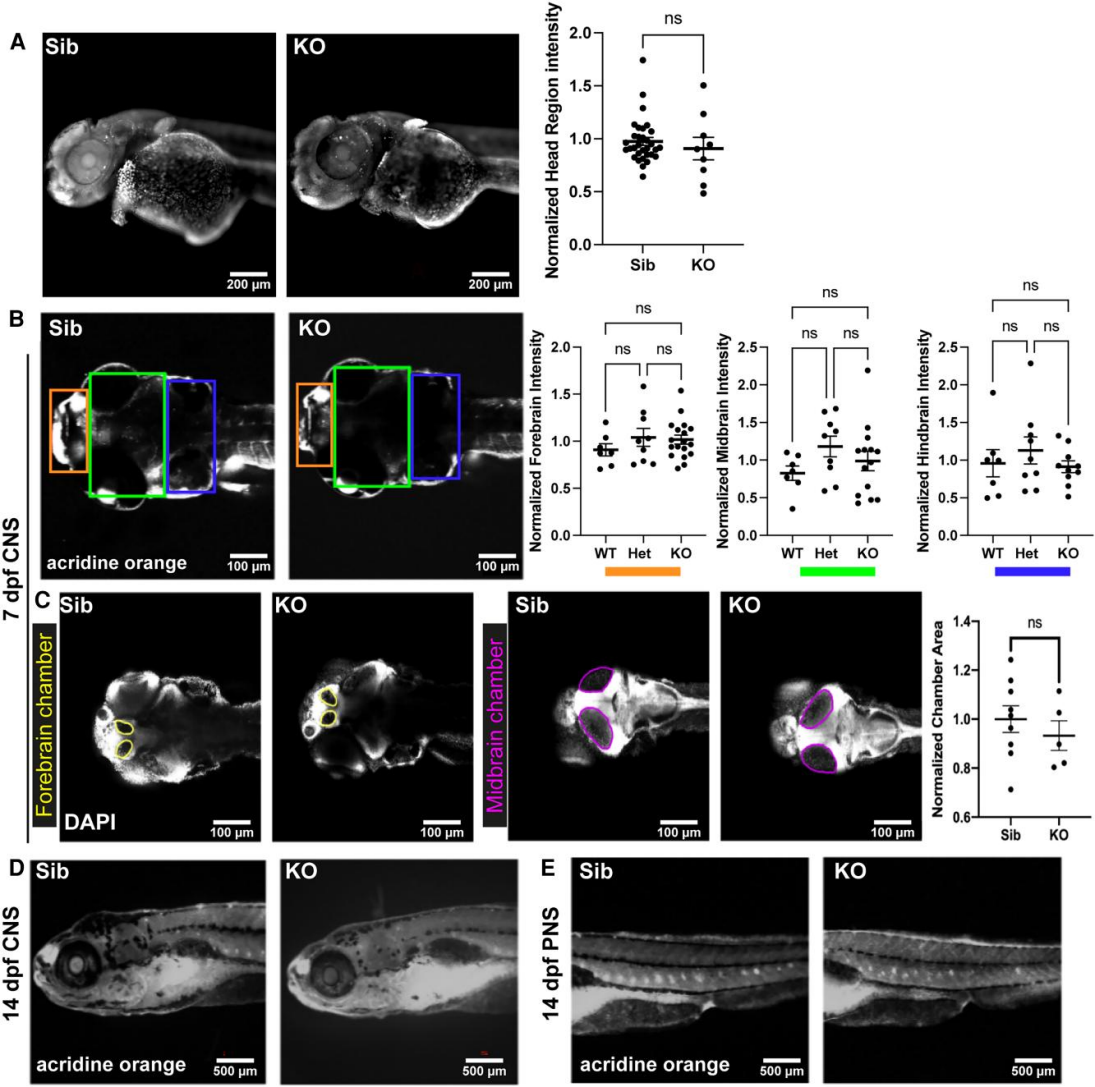
**Loss of *mtmr5* does not result in excessive cell death in the nervous system and does not cause changes in brain chamber size.** (**A** and **B**) Representative average Z-projections depicting laterally orientated 2 days post-fertilization (dpf) (**A**) and 7 dpf (**B**) zebrafish embryos stained with acridine orange (AO) to visualize and quantify apoptotic cells in live zebrafish brains. No differences in AO intensity were detected between the sibling and the knockout (KO) (**A**—2 dpf: Sib *n* = 32, KO *n* = 9; **B**—7 dpf: WT *n* = 7, Het *n* = 9, KO *n* = 18). (**C**) Representative images from fixed and DAPI-stained 7 dpf zebrafish embryos to visualize and quantify brain chamber size. There were no qualitative or quantitative differences in both forebrain and midbrain size between the siblings and KOs at 7 dpf (Sib *n* = 9, KO n = 5). (**D** and **E**) Representative images of AO-stained (**D**) anterior lateral regions of 14 dpf zebrafish, encompassing the central nervous system, show no visual differences between the siblings and KOs, and (**E**) posterior-lateral regions of 14 dpf zebrafish, encompassing the peripheral nervous system, show no visual differences between the siblings and KOs. All statistical analyses include at least three independent experiments. Each dot on the graphs represents one zebrafish, at least *n* = 3 zebrafish were used per group per experiment. Quantitative data are mean±SEM, normalized to the average of WT and presented as ratios. Unpaired two-tailed Student’s *t*-test was used. ns, not significant. Scale bars: 200 μm (**A**), 100 μm (**B** and **C**) or 500 μm (**D**).

We further examined the microcephaly phenotype to determine if it was due to excessive neuron cell death or loss of brain volume. To study apoptosis in the nervous system, we performed acridine orange (AO) staining on live zebrafish at 2 dpf (Fig. 5A), 7 dpf (Fig. 5B) and 14 dpf (Fig. 5C and D). We observed no accumulation of signal, and no increase in number of AO positive cells, in either the head (CNS, central nervous system) or in the trunk (PNS, peripheral nervous system) of *mtmr5*-KO zebrafish. To visualize the ventricular system, we performed DAPI staining on fixed 7 dpf zebrafish (Fig. 5B, where DAPI negative areas represent the ventricles), and detected no differences between WT sibling and *mtmr5*-KO in the size of any brain ventricle chamber. In other words, knockout of *mtmr5* caused no enlargement in brain ventricle size, which might be expected if there was brain atrophy due to brain cell loss. Also, we examined autophagy by performing Western blotting to compare LC3 levels in WT versus *mtmr5*-KO brains and observed no significant difference (Supplementary Fig. 3). In total, these data demonstrate that the microcephaly phenotype in *mtmr5-*KO zebrafish is not caused by excessive apoptosis, autophagy or cell loss in the nervous system, indicating instead potential defects in early neurogenesis.

### Loss of *mtmr5* induced axon defects and dysmyelination in the developing nervous system

**Figure 6:**
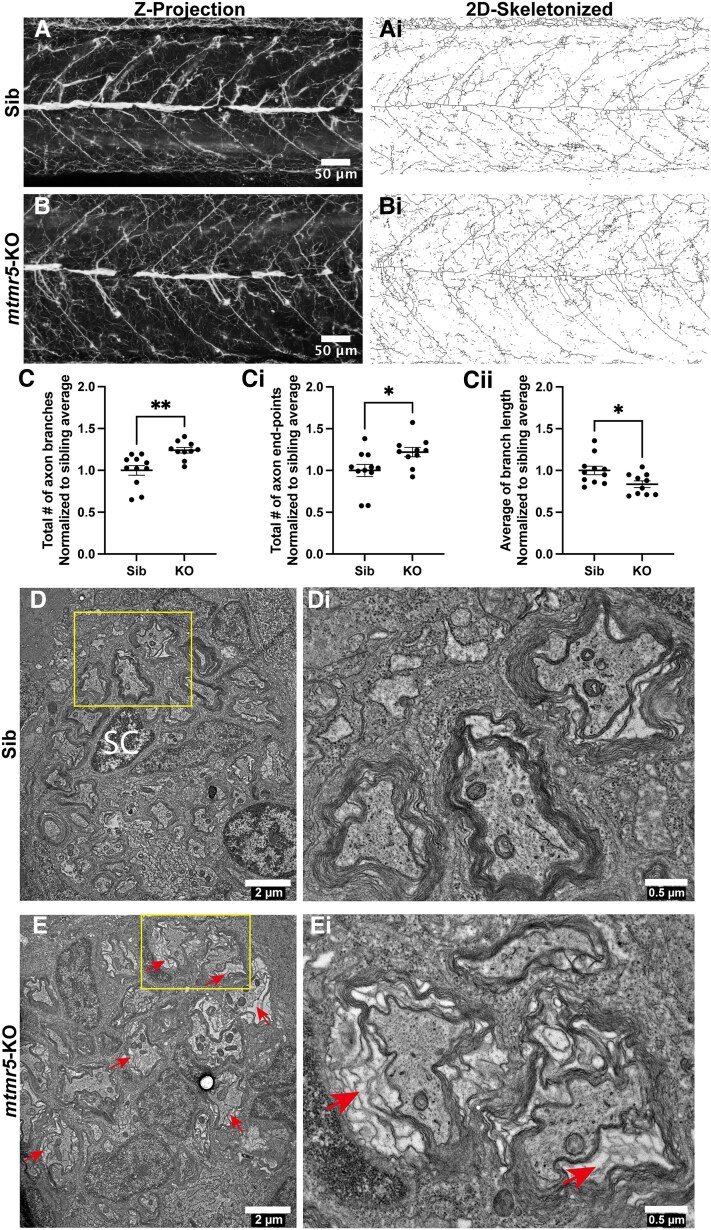
**Loss of *mtmr5* leads to less defined axonal organization and dysregulated axon myelination.** (**A** and **B**) Seven days post-fertilization (dpf) zebrafish embryos were fixed and probed with an acetylated tubulin antibody to label mature axons. (**A** and **B**) Representative images of *Z*-projections (standard deviation via Fiji ImageJ) of acetylated tubulin staining. Axons appear less organized in the *mtmr5*-knockout (KO) compared to the sibling. (**Ai** and **Bi**) Two-dimensional skeletonized images generated using Binary function in Fiji ImageJ to allow for quantification of axonal organizational parameters. (**C-Cii**) The number of total axonal branches (**C**) and axon end-points (**Ci**) significantly increased (by ∼25%) in the KO compared to the siblings (Sib), while the average axon branch length (**Cii**) in KOs significantly decreased (by ∼10%). All quantification was normalized to the sibling average of the respective parameter. All statistical analyses include at least three independent experiments. Each dot on the graphs represents one zebrafish. Quantitative data are mean±SEM, normalized to the average of WT and presented as ratios. Unpaired two-tailed Student’s *t*-test was used. *P < 0.05; **P < 0.01, ns, not significant. Scale bars (**A** and **B**): 20 μm. Sib *n* = 11, KO *n* = 10. (**D**) Transmission electron microscopy was used to visualize axons in the posterior lateral line at 14 dpf. WT axons were in close contact with their myelin sheaths. This tight and organized wrapping (**D**, box) can be observed in more detail in the zoomed-in inset (**Di**). SC = Schwann cell. (**E**) In the 14 dpf KO, the myelin is still present; yet, there is an apparent blank space between the axon and the myelin (arrows), showing the detachment or loose myelin in the KOs (**E**, yellow box; **Ei**). Scale bars: (**D** and **E**) 2 μm or (**Di** and **Ei**) 0.5 μm.

CMT4B3 patients show both axon and myelination defects in the peripheral nervous system.^9,24,25^ In zebrafish, while all major components of the brain are present by 5 dpf, primary motor neurons continue to develop until 30 dpf, and the lateral line (composed of peripheral sensory neurons) is not fully developed until 2 months.^37^ To examine axon patterning in *mtmr5*-KO, we performed whole mount immunofluorescence on 7 dpf zebrafish larvae using an antibody against acetylated alpha tubulin, which labels mature axons. Interestingly, confocal images showed disorganized and less defined axon morphology in the *mtmr5-*KOs (Fig. 6A and B). To quantify axon branching, we utilized the Skeleton plugin in Fiji ImageJ (Fig. 6Ai and Bi), which measures parameters such as the number of axon branches, the number of end-points (where branches end), and the length of axon branches. We detected a significant upregulation of the total number of branches (Fig. 6C) and the total number of end-points (Fig. 6Ci) in the *mtmr5-*KOs, while the average branch length was significantly reduced (Fig. 6Cii). These data support that *mtmr5*-KO disrupts axonal patterning and morphogenesis during neural development.

To examine PNS myelination in the *mtmr5*-KO zebrafish, we imaged transverse sections of WT versus *mtmr5*-KO posterior lateral line (a sensory system in zebrafish to detect water flow) under transmission electron microscopy. In 14 dpf WT siblings, most axons were in close proximity to the myelin sheaths (Fig. 6D-Di). In the *mtmr5*-KO, we did not observe lack of myelination; however, we did observe an increased number of axons showing plasma membrane detachment from the myelin sheaths (Fig. 6E and Ei), consistent with a dysmyelination phenotype.

### Transcriptomic analysis shows dysregulation in neurogenesis, chromatin organization, and synaptic biogenesis in 7 dpf *mtmr5*-KO zebrafish brain-enriched samples

**Figure 7:**
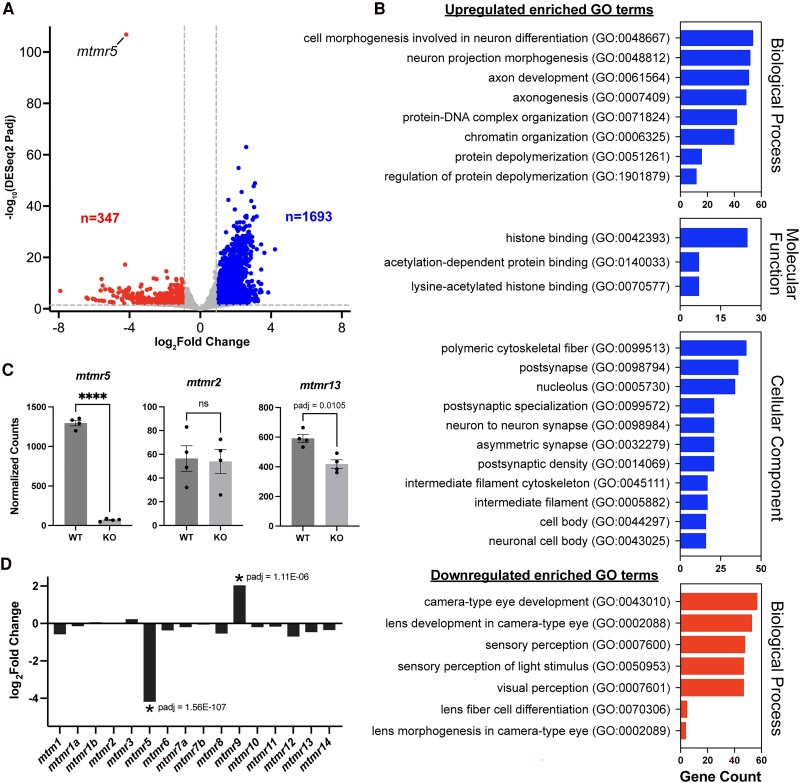
**Bulk RNA sequencing of 7 dpf *mtmr5*-knockout versus wild-type brain-enriched samples.** (**A**) Volcano plot displaying the differentially expressed genes in 7 days-post-fertilization (dpf) *mtmr5*-knockout (KO) zebrafish heads compared to wild-type (WT) siblings. A significance threshold of adjusted *P*-value (padj) < 0.01 and absolute log_2_ fold-change >1 was used. Upregulated genes are in the top right segment and downregulated genes are in top left segment. Number of significant genes in each category are indicated (upregulated, *n* = 1693; downregulated, *n* = 347). Each dot represents one zebrafish gene. See Supplementary Table 2 for the full list of genes, padj values, and log_2_fold-changes. (**B**) Gene ontology (GO) terms significantly enriched in the up- and down-regulated genes (padj < 0.01), sorted by the number of genes associated with the respective GO term (gene count). All enriched GO terms (padj < 0.05) are included in Supplementary Table 3. (**C**) Normalized read counts for the three CMT4B-associated MTMR genes: *mtmr5*, *mtmr2* and *mtmr13*. ****padj < 0.00001, ns > 0.05. (**D**) Log_2_ fold-change values for all *mtmr* genes with significant values indicated (padj < 0.01). *mtmr5* and *mtmr13* gene names are used in place of *sbf1* and *sbf2*, respectively.

To identify MTMR5-associated disease pathways, we performed RNA sequencing using brain-enriched RNAs isolated from WT and *mtmr5*-KO zebrafish heads at 7 dpf, i.e. before the onset of significant brain size reduction in the KO. Raw sequencing data were analyzed to produce a comprehensive list of zebrafish genes and their relative expression level changes in *mtmr5*-KOs compared to WT siblings (Supplementary Table 2). Principal component analysis (PCA) revealed independent clustering of WT and *mtmr5*-KO replicate samples, indicating distinct transcriptomic profiles between the two conditions (Supplementary Fig. 4). At a significance threshold of adjusted *P*-value (padj) < 0.01 and absolute log2fold-change >1, 2040 genes were identified as being differentially expressed in the *mtmr5*-KOs: 1693 upregulated and 347 downregulated (Fig. 7A). Upregulated genes were enriched in gene ontology (GO) pathways relating to neurogenesis, chromatin organization/remodelling, cytoskeletal fibre polymerization, and synaptic signalling (Fig. 7B). Downregulated genes were enriched in GO terms relating to eye development (despite no overt ocular changes) and protein translation (Supplemental Table 3). Furthermore, we looked at all proteins in the myotubularin family in WT versus *mtmr5*-KO; as predicted, *mtmr5* is detected in the 7 dpf WT brain, but essentially absent in the KO (Fig. 7C). *mtmr5*-KO also showed a ∼25% downregulation of *mtmr13* expression (*P*adj = 0.0105), no change in the level of *mtmr2*, and upregulation in *mtmr9*, a catalytically inactive MTMR also involved in autophagy suppression (Fig. 7D).^38^

## Discussion

CMT4B3 is an early-onset neuropathy that currently has no treatment. It is caused by recessive pathogenic variants in the *MTMR5* gene. In this study, we report the first *mtmr5* zebrafish knockout line generated by CRISPR/Cas9-mediated gene editing. *mtmr5* is predominantly expressed in the brain in zebrafish, and consistent with this, loss of *mtmr5* in zebrafish results in early-onset microcephaly without affecting overall survival or motor performance. Of note, brain/CNS involvement is a disease hallmark of CMT4B3 patients that has not been reported in other CMT4B subtypes or in the mouse models of *MTMR5*. In addition, upon examining phenotypes in the peripheral nervous system (PNS), we observed disrupted axon patterning and dysmyelination in *mtmr5-*KO zebrafish, similar to the polyneuropathy reported in patients.^25-27^ Moreover, RNA sequencing using brain-enriched zebrafish cDNA identified changes in novel pathways (e.g. chromatin remodelling and synaptic biogenesis) that will guide future studies into the pathomechanisms underlying MTMR5-related brain abnormalities. Our zebrafish model is thus a first *MTMR5* animal model that recapitulates multiple aspects of the clinical spectrum of CMT4B3 disease.

MTMR5 has been known as a pseudophosphatase that binds to its active family member MTMR2.^12^ MTMR2 subcellular localization is either diffuse or cytoplasm-specific depending on the cell type^1^ and its interaction with MTMR5.^6^ MTMR5 thereby may function (at least partially) by recruiting MTMR2 to its substrates and promoting its enzymatic activity.^6^ Notably, CMT4B1-causing *MTMR2* mutations drastically reduce its phosphatase activity,^39^ suggesting the importance of MTMR2 activity in ensuring normal PNS development/function. In our *mtmr5*-KO zebrafish, we see a near complete absence of *mtmr5* transcript without changes in *mtmr2* levels, although we were not able to evaluate Mtmr2/5/13 protein expression or localization in the zebrafish PNS or CNS (due to lack of suitable antibodies). Given the transcript reduction and the nature of the mutation (full gene deletion), we predict that no Mtmr5 protein is present in our mutant fish line. Future studies (such as examination of transgenic zebrafish with tagged MTMR2/5/13) will be required to determine normal Mtmr5 localization in neuronal cell types and if Mtmr2/13 subcellular localization/function is altered in the *mtmr5*-KO nervous system. Notably, each form of CMT4B is associated with distinct features in patients and distinct PNS nerve pathologies in mouse models: *Mtmr2* −/− and *Mtmr13 −/−* mice showed classic CMT4B myelin outfolding,^3,40,41^ whilst adult *Mtmr5 −/−* mice showed reduced number of myelinated axons likely due to disruption in axon radial sorting.^12^ These data, in combination with our findings in the zebrafish, suggest that MTMR5 may have additional roles beyond those specifically related to MTMR2 and MTMR13.

In the zebrafish, we observed *mtmr5* expression restricted to the CNS from 1 to 3 dpf, and the onset of microcephaly phenotype in >10 dpf *mtmr5*-KO zebrafish larvae, supporting the expanded CNS phenotype in CMT4B3 patients, and a novel role of MTMR5 in CNS development. Due to the absence of excessive cell death, the microcephaly observed in *mtmr5*-KO zebrafish is likely a result of developmental abnormalities/delays rather than neurodegeneration. Moreover, a recent report identified MTMR5 association with Alzheimer’s disease in aged population,^42^ indicating MTMR5 may also be a genetic modifier in CNS maintenance. At present, though, little is known about the brain cell population/function of MTMR5, or its subcellular localization/function within neural cells. Surprisingly, our brain-enriched transcriptomic analysis suggests that MTMR5 may regulate chromatin remodelling/organization pathway in the brain, mirroring our recent findings of epigenetic changes associated with knockout of another myotubularin family member (MTM1) in skeletal muscle.^43^ Future work is needed to unravel the role(s) of MTMR5 in neurons and brain development and establish why its loss causes microcephaly. Multi-omic platforms, such as spatial transcriptomics and proximity labeling with BioID, are potential approaches to unbiasedly identify cellular populations and pathways associated with MTMR5.

Of note, our data as well illuminates PNS pathology of axon patterning and myelination in larval stage zebrafish. Future studies will focus on tracing these abnormalities to their onset, and determining when (and in what cell type(s)) during embryogenesis these changes first occur. Use of differentially expressed ‘marker’ genes (e.g. nrg1 and mbpa), identified through our RNA sequencing analysis, could enable detection of early changes in Schwann cell development and maturation and also axonogenesis, as could examination of classical markers of different aspects of the PNS differentiation process including using live imaging to study axon myelination *in vivo.*^44^ In terms of our RNA sequencing study, the DEGs identified provide an exciting first description in the molecular characterization of *mtmr5*-KO pathogenesis. Follow-up experimentation will be needed to verify these changes, with examination at the protein level (via antibodies when possible or tagged transgenic lines) as a key next step.

Interestingly, whilst molecular and structural phenotypes are detected in both the CNS and PNS, *mtmr5*-KO zebrafish display no overt deficits in motor function, consistent with observations in adult *Mtmr5*-KO mice. The possibility of genetic compensation, which has been identified as an important cause of reduced phenotypic expression in other CRISPR generated zebrafish mutants,^45,46^ may explain the absence of a severe motor phenotype in our *mtmr5-*KO zebrafish larvae. However, this is most commonly described in small coding sequence ‘indel’ mutants,^46^ and our generation of a full gene deletion mutation was done in part to avoid/prevent secondary compensation. There is of course the potential for disease modification in zebrafish by other related gene products, such as for example the upregulation of *mtmr9* as detected by our brain-enriched RNA sequencing. Future experiments designed at targeting potential modifier genes in the *mtmr5*-KO background will help to better clarify this. In addition, the potential for environmental factors (e.g. chemical/mechanical stress) to induce motor phenotypes can be explored.

Lastly, we identified the microcephaly present at 10 dpf as the most feasible (and disease relevant) phenotype for future therapy identification and development. Zebrafish serve as an excellent model for drug screens due to their high gene conservation with human and ease of drug administration.^17^ While microcephaly as an outcome would be somewhat low throughput and likely not amenable to unbiased screens with 1000s of compounds, it should be suitable to evaluate dozens to 100s of candidate target compounds. Moreover, differentially expressed genes can be leveraged as quantifiable phenotypes, e.g. by tagging their promoters with GFP, to enable fluorescence-based drug screening. Candidate treatments to test include drugs previously shown to work in other CMT subtypes,^47,48^ other MTMR-related neuromuscular disorders (e.g. valproic acid,^43^ tamoxifen^49^ and wortmannin^50^ in X-linked myotubular myopathy due to *MTM1* mutation), and drug libraries targeting MTMR5-related pathways, for example, epigenetics (this study), endocytosis and autophagy.^51^

In conclusion, we have established the *mtmr5-*KO zebrafish as a versatile preclinical model for elucidating CMT4B3 pathomechanisms and advancing therapy development.

## Supplementary Material

fcaf077_Supplementary_Data

## Data Availability

All data relevant to the study are provided in the manuscript. RNA-sequencing data is available in the Gene Expression Omnibus (https://www.ncbi.nlm.nih.gov/geo/). Source images and data are stored at the Hospital for Sick Children and are available upon request.

## References

[1] Previtali SC, Quattrini A, Bolino A. Charcot–Marie–Tooth type 4B demyelinating neuropathy: Deciphering the role of MTMR phosphatases. Expert Rev Mol Med. 2007;9(25):1–16. 10.1017/S1462399407000439 17880751

[2] Bolino A, Bolis A, Previtali SC, et al Disruption of Mtmr2 produces CMT4B1-like neuropathy with myelin outfolding and impaired spermatogenesis. J Cell Biol. 2004;167(4):711–721. 15557122 10.1083/jcb.200407010 PMC2172586

[3] Tersar K, Boentert M, Berger P, et al Mtmr13/Sbf2-deficient mice: An animal model for CMT4B2. Hum Mol Genet. 2007;16(24):2991–3001. 17855448 10.1093/hmg/ddm257

[4] Nakhro K, Park J-M, Hong YB, et al SET binding factor 1 (SBF1) mutation causes Charcot-Marie-Tooth disease type 4B3. Neurology. 2013;81(2):165–173. 23749797 10.1212/WNL.0b013e31829a3421

[5] Bolis A, Zordan P, Coviello S, Bolino A. Myotubularin-related (MTMR) phospholipid phosphatase proteins in the peripheral nervous system. Mol Neurobiol. 2007;35(3):308–316. 17917119 10.1007/s12035-007-0031-0

[6] Kim S-A, Vacratsis PO, Firestein R, Cleary ML, Dixon JE. Regulation of myotubularin-related (MTMR)2 phosphatidylinositol phosphatase by MTMR5, a catalytically inactive phosphatase. Proc Natl Acad Sci U S A. 2003;100(8):4492–4497. 12668758 10.1073/pnas.0431052100 PMC153583

[7] Robinson FL, Dixon JE. The phosphoinositide-3-phosphatase MTMR2 associates with MTMR13, a membrane-associated pseudophosphatase also mutated in type 4B Charcot-Marie-Tooth disease. J Biol Chem. 2005;280(36):31699–31707. 15998640 10.1074/jbc.M505159200

[8] Gang Q, Bettencourt C, Holton J, et al A novel frameshift deletion in autosomal recessive SBF1-related syndromic neuropathy with necklace fibres. J Neurol. 2020;267(9):2705–2712. 32444983 10.1007/s00415-020-09827-y PMC7419361

[9] Romani M, Mehawej C, Mazza T, Mégarbané A, Valente EM. Fork and bracket” syndrome expands the spectrum of SBF1-related sensory motor polyneuropathies. Neurol Genet. 2016;2(2):e61. 27123480 10.1212/NXG.0000000000000061 PMC4830194

[10] Berti B, Longo G, Mari F, et al Bi-allelic variants in MTMR5/SBF1 cause Charcot-Marie-Tooth type 4B3 featuring mitochondrial dysfunction. BMC Med Genomics. 2021;14(1):157. 34118926 10.1186/s12920-021-01001-1 PMC8199524

[11] Firestein R, Nagy PL, Daly M, Huie P, Conti M, Cleary ML. Male infertility, impaired spermatogenesis, and azoospermia in mice deficient for the pseudophosphatase Sbf1. J Clin Invest. 2002;109(9):1165–1172. 11994405 10.1172/JCI12589 PMC150957

[12] Mammel AE, Delgado KC, Chin AL, et al Distinct roles for the Charcot–Marie–Tooth disease-causing endosomal regulators Mtmr5 and Mtmr13 in axon radial sorting and Schwann cell myelination. Hum Mol Genet. 2022;31(8):1216–1229. 34718573 10.1093/hmg/ddab311 PMC9190308

[13] Santoriello C, Zon LI. Hooked! modeling human disease in zebrafish. J Clin Invest. 2012;122(7):2337–2343. 22751109 10.1172/JCI60434 PMC3386812

[14] Reddy H, Chapman AL, Bennett EJ, Ramesh TM, De Vos KJ, Grierson AJ. Axonal transport defects in a mitofusin 2 loss of function model of Charcot-Marie-Tooth disease in zebrafish. PLoS One. 2013;8(6):e67276. 23840650 10.1371/journal.pone.0067276 PMC3694133

[15] Dowling JJ, Low SE, Busta AS, Feldman EL. Zebrafish MTMR14 is required for excitation–contraction coupling, developmental motor function and the regulation of autophagy. Hum Mol Genet. 2010;19(13):2668–2681. 20400459 10.1093/hmg/ddq153 PMC2883342

[16] Preston MA, Macklin WB. Zebrafish as a model to investigate CNS myelination. Glia. 2014;63(2):177–193. 25263121 10.1002/glia.22755 PMC4539269

[17] Karuppasamy M, English KG, Henry CA, et al Standardization of zebrafish drug testing parameters for muscle diseases. Dis Model Mech. 2024;17(1):dmm050339. 38235578 10.1242/dmm.050339 PMC10820820

[18] Letunic I, Khedkar S, Bork P. SMART: Recent updates, new developments and status in 2020. Nucleic Acids Res. 2021;49(D1):D458–D460. 33104802 10.1093/nar/gkaa937 PMC7778883

[19] Ludwiczak J, Winski A, Szczepaniak K, Alva V, Dunin-Horkawicz S, Hancock J. DeepCoil—A fast and accurate prediction of coiled-coil domains in protein sequences. Bioinformatics. 2019;35(16):2790–2795. 30601942 10.1093/bioinformatics/bty1062

[20] Xie Y, Li H, Luo X, et al IBS 2.0: An upgraded illustrator for the visualization of biological sequences. Nucleic Acids Res. 2022;50(W1):W420–W426. 35580044 10.1093/nar/gkac373 PMC9252815

[21] Marat AL, Dokainish H, McPherson PS. DENN domain proteins: Regulators of Rab GTPases. J Biol Chem. 2011;286(16):13791–13800. 21330364 10.1074/jbc.R110.217067 PMC3077579

[22] Zhang D, Iyer LM, He F, Aravind L. Discovery of novel DENN proteins: Implications for the evolution of eukaryotic intracellular membrane structures and human disease. Front Genet. 2012;3:283. 23248642 10.3389/fgene.2012.00283 PMC3521125

[23] Laporte J, Bedez F, Bolino A, Mandel JL. Myotubularins, a large disease-associated family of cooperating catalytically active and inactive phosphoinositides phosphatases. Hum Mol Genet. 2003;12(suppl 2):R285–R292. 12925573 10.1093/hmg/ddg273

[24] Alazami AM, Alzahrani F, Bohlega S, Alkuraya FS. SET binding factor 1 (SBF1) mutation causes Charcot-Marie-Tooth disease type 4B3. Neurology. 2014;82(18):1665–1666. 24799518 10.1212/WNL.0000000000000331

[25] Flusser H, Halperin D, Kadir R, Shorer Z, Shelef I, Birk OS. Novel SBF1 splice-site null mutation broadens the clinical spectrum of Charcot-Marie-Tooth type 4B3 disease. Clin Genet. 2018;94(5):473–479. 30039846 10.1111/cge.13419

[26] Manole A, Horga A, Gamez J, et al SBF1 mutations associated with autosomal recessive axonal neuropathy with cranial nerve involvement. Neurogenetics. 2017;18(1):63–67. 28005197 10.1007/s10048-016-0505-1

[27] Mégarbané A, Dorison N, Rodriguez D, Tamraz J. Multiple cranial nerve neuropathies, microcephaly, neurological degeneration, and “fork and bracket sign” in the MRI: A distinct syndrome. Am J Med Genet A. 2010;152A(9):2297–2300. 20658556 10.1002/ajmg.a.33417

[28] Landrum MJ, Chitipiralla S, Brown GR, et al ClinVar: Improvements to accessing data. Nucleic Acids Res. 2020;48(D1):D835–D844. 31777943 10.1093/nar/gkz972 PMC6943040

[29] Thisse C, Thisse B. High-resolution in situ hybridization to whole-mount zebrafish embryos. Nat Protoc. 2007;3(1):59–69. 10.1038/nprot.2007.514 18193022

[30] Montague TG, Cruz JM, Gagnon JA, Church GM, Valen E. CHOPCHOP: A CRISPR/Cas9 and TALEN web tool for genome editing. Nucleic Acids Res. 2014;42 (Web Server issue):W401–W407. 24861617 10.1093/nar/gku410 PMC4086086

[31] Espinosa KG, Geissah S, Groom L, et al Characterization of a novel zebrafish model of SPEG-related centronuclear myopathy. Dis Model Mech. 2022;15(5):dmm049437. 35293586 10.1242/dmm.049437 PMC9118044

[32] Zhao M, Smith L, Volpatti J, Fabian L, Dowling JJ. Insights into wild-type dynamin 2 and the consequences of DNM2 mutations from transgenic zebrafish. Hum Mol Genet. 2019;28(24):4186–4196. 31691805 10.1093/hmg/ddz260

[33] Fabian L, Karimi E, Farman GP, et al Comprehensive phenotypic characterization of an allelic series of zebrafish models of NEB-related nemaline myopathy. Hum Mol Genet. 2024;33(12):1036–1054. 38493359 10.1093/hmg/ddae033 PMC11153343

[34] Abueg Linelle Ann L, Afgan E, Allart O, et al The Galaxy platform for accessible, reproducible, and collaborative data analyses: 2024 update. Nucleic Acids Res. 2024;52(W1):W83–W94. 38769056 10.1093/nar/gkae410 PMC11223835

[35] Love Michael I, Huber W, Anders S. Moderated estimation of fold change and dispersion for RNA-seq data with DESeq2. Genome Biol. 2014;15(12). 10.1186/s13059-014-0550-8 PMC4302049 25516281

[36] Wu T, Hu E, Xu S, et al clusterProfiler 4.0: A universal enrichment tool for interpreting omics data. The Innovation. 2021;2(3):100141. 34557778 10.1016/j.xinn.2021.100141 PMC8454663

[37] Cunliffe VT . Zebrafish: A practical approach. Edited by C. NÜSSLEIN-VOLHARD and R. DAHM. Oxford University Press. 2002. 322 pages. ISBN 0 19 963808 X. Price £40.00 (paperback). ISBN 0 19 963809 8. Price £80.00 (hardback). Genet Res. 2003;82(1):79.

[38] Wang J, Guo W, Wang Q, Yang Y, Sun X. Recent advances of myotubularin-related (MTMR) protein family in cardiovascular diseases. Front Cardiovasc Med. 2024;11:1364604. 38529329 10.3389/fcvm.2024.1364604 PMC10961392

[39] Berger P, Schaffitzel C, Berger I, Ban N, Suter U. Membrane association of myotubularin-related protein 2 is mediated by a pleckstrin homology-GRAM domain and a coiled-coil dimerization module. Proc Natl Acad Sci U S A. 2003;100(21):12177–12182. 14530412 10.1073/pnas.2132732100 PMC218732

[40] Robinson FL, Niesman IR, Beiswenger KK, Dixon JE. Loss of the inactive myotubularin-related phosphatase Mtmr13 leads to a Charcot–Marie–Tooth 4B2-like peripheral neuropathy in mice. Proc Natl Acad Sci U S A. 2008;105(12):4916–4921. 18349142 10.1073/pnas.0800742105 PMC2290800

[41] Azzedine H, Bolino A, Taïeb T, et al Mutations in MTMR13, a new pseudophosphatase homologue of MTMR2 and sbf1, in two families with an autosomal recessive demyelinating form of Charcot-Marie-Tooth disease associated with early-onset glaucoma. Am J Hum Genet. 2003;72(5):1141–1153. 12687498 10.1086/375034 PMC1180267

[42] Khamse S, Alizadeh S, Bernhart SH, Afshar H, Delbari A, Ohadi M. A (GCC) repeat in SBF1 reveals a novel biological phenomenon in human and links to late onset neurocognitive disorder. Sci Rep. 2022;12(1):15480. 36104480 10.1038/s41598-022-19878-y PMC9474449

[43] Volpatti JR, Ghahramani-Seno MM, Mansat M, et al X-linked myotubular myopathy is associated with epigenetic alterations and is ameliorated by HDAC inhibition. Acta Neuropathol. 2022;144(3):537–563. 35844027 10.1007/s00401-022-02468-7 PMC9381459

[44] Almeida RG, Czopka T, ffrench-Constant C, Lyons DA. Individual axons regulate the myelinating potential of single oligodendrocytes in vivo. Development. 2011;138(20):4443–4450. 21880787 10.1242/dev.071001 PMC3177314

[45] Moens C, El-Brolosy MA, Stainier DYR. Genetic compensation: A phenomenon in search of mechanisms. PLoS Genet. 2017;13(7):e1006780. 28704371 10.1371/journal.pgen.1006780 PMC5509088

[46] Rossi A, Kontarakis Z, Gerri C, et al Genetic compensation induced by deleterious mutations but not gene knockdowns. Nature. 2015;524(7564):230–233. 26168398 10.1038/nature14580

[47] Stavrou M, Sargiannidou I, Georgiou E, Kagiava A, Kleopa KA. Emerging therapies for Charcot-Marie-Tooth inherited neuropathies. Int J Mol Sci. 2021;22(11):6048. 34205075 10.3390/ijms22116048 PMC8199910

[48] Pisciotta C, Saveri P, Pareyson D. Challenges in treating Charcot-Marie-Tooth disease and related neuropathies: Current management and future perspectives. Brain Sci. 2021;11(11):1447. 34827446 10.3390/brainsci11111447 PMC8615778

[49] Maani N, Sabha N, Rezai K, et al Tamoxifen therapy in a murine model of myotubular myopathy. Nat Commun. 2018;9(1):4849. 30451841 10.1038/s41467-018-07057-5 PMC6242823

[50] Sabha N, Volpatti JR, Gonorazky H, et al PIK3C2B inhibition improves function and prolongs survival in myotubular myopathy animal models. J Clin Invest. 2016;126(9):3613–3625. 27548528 10.1172/JCI86841 PMC5004942

[51] Chua JP, Bedi K, Paulsen MT, et al Myotubularin-related phosphatase 5 is a critical determinant of autophagy in neurons. Curr Biol. 2022;32(12):2581–2595.e6. 35580604 10.1016/j.cub.2022.04.053 PMC9233098

