# Supplementary material for: Characterization of a novel zebrafish model of MTMR5-associated Charcot-Marie-Tooth disease type 4B3
Source: Brain Commun. 2025 Feb 18;7(2):fcaf077. doi: 10.1093/braincomms/fcaf077 (PMC11891516; doi:10.1093/braincomms/fcaf077)
Supplement: fcaf077_Supplementary_Data [file fcaf077_supplementary_data.zip › Supplementary figures.pdf]

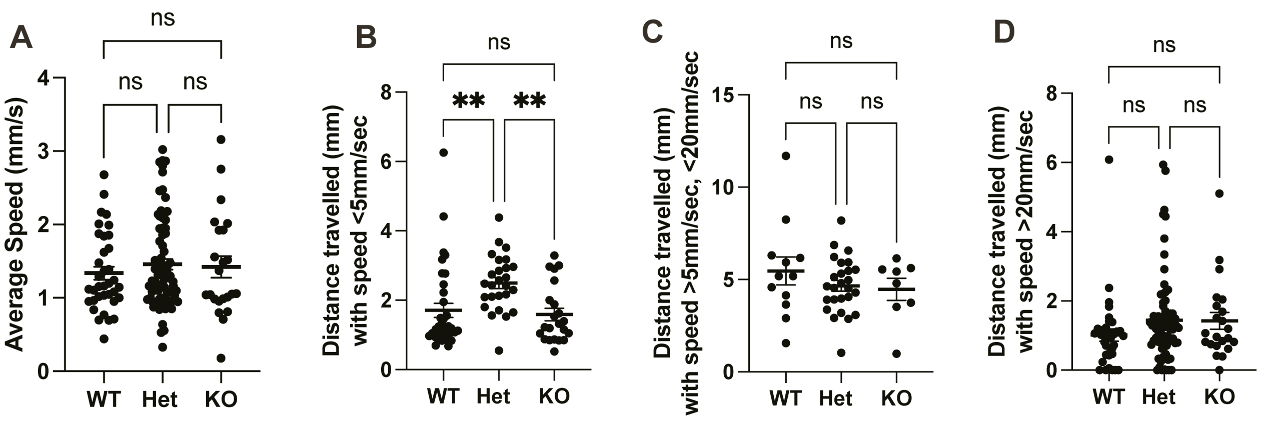

Supplementary Figure 1. Loss of *mtmr5* does not affect gross motor functions (other parameters). (A) Embryos generated from a *mtmr5*-heterozygous in-cross were subjected to Optovin treatment at 3 days-post-fertilization (dpf) followed by the quantification of the average speed (mm/s) by each embryo via Zebrabox. There was no apparent difference in speed between wild-type (WT); n= 26, heterozygous (Het); = 22, and knockouts (KO); n=22. No significant difference was detected in the small (B), intermediate (C) or large (D) locomotion levels from the same experiments, except for an increase in heterozygous distance travelled (speed<5mm/sec) compared to WT and KOs. All statistical analyses include at least three independent experiments. Each dot on the graphs represents one zebrafish embryo, at least n=4 embryos were used per group per experiment. Data are mean ± SEM. All measurements are in millimeters (mm). One-way ANOVA was used: **P<0.01; ns, not significant.

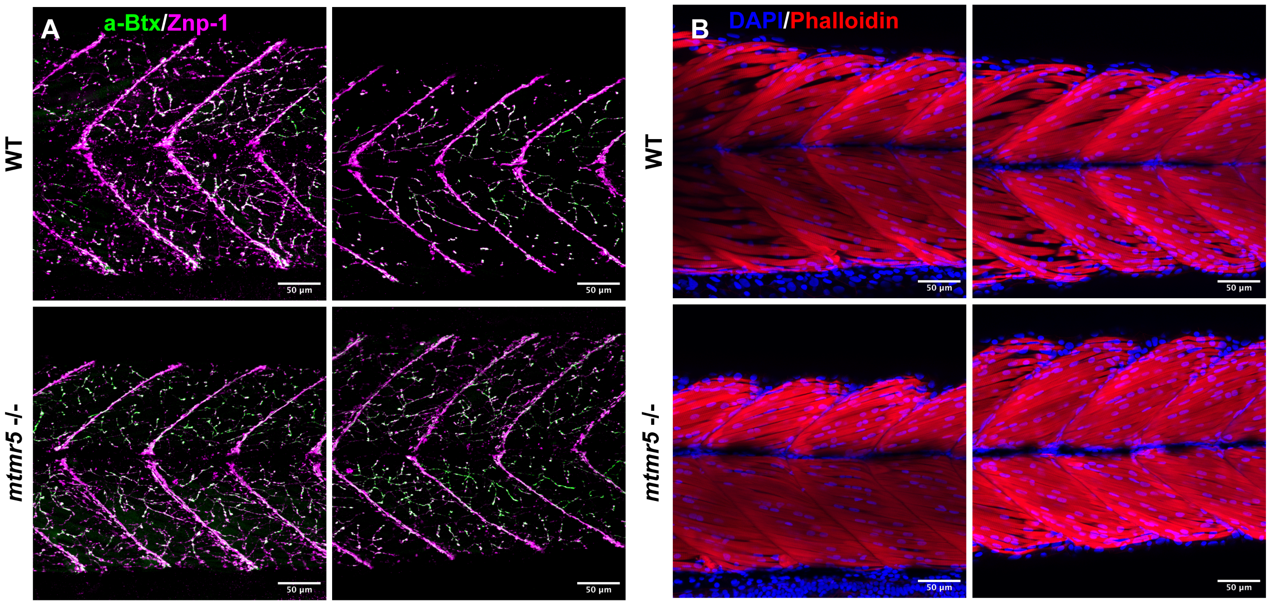

Supplementary Figure 2. Whole-mount immunofluorescence showed normal synapse and sarcomeric integrity in 7 dpf *mtmr5* -/- zebrafish larvae. (A) *mtmr5*-KO larvae showed normal pre-synapse (Znp-1, magenta) and post-synapse (alpha-Bungarotoxin, green) organization, as well as pre-/post-synapse overlapping in the peripheral nervous system. (B) *mtmr5*-KO larvae showed normal filamentous actin (phalloidin, red) or thin filament organization in the myotomes. DAPI (blue, nucleus). Scale bars: 50 μm.

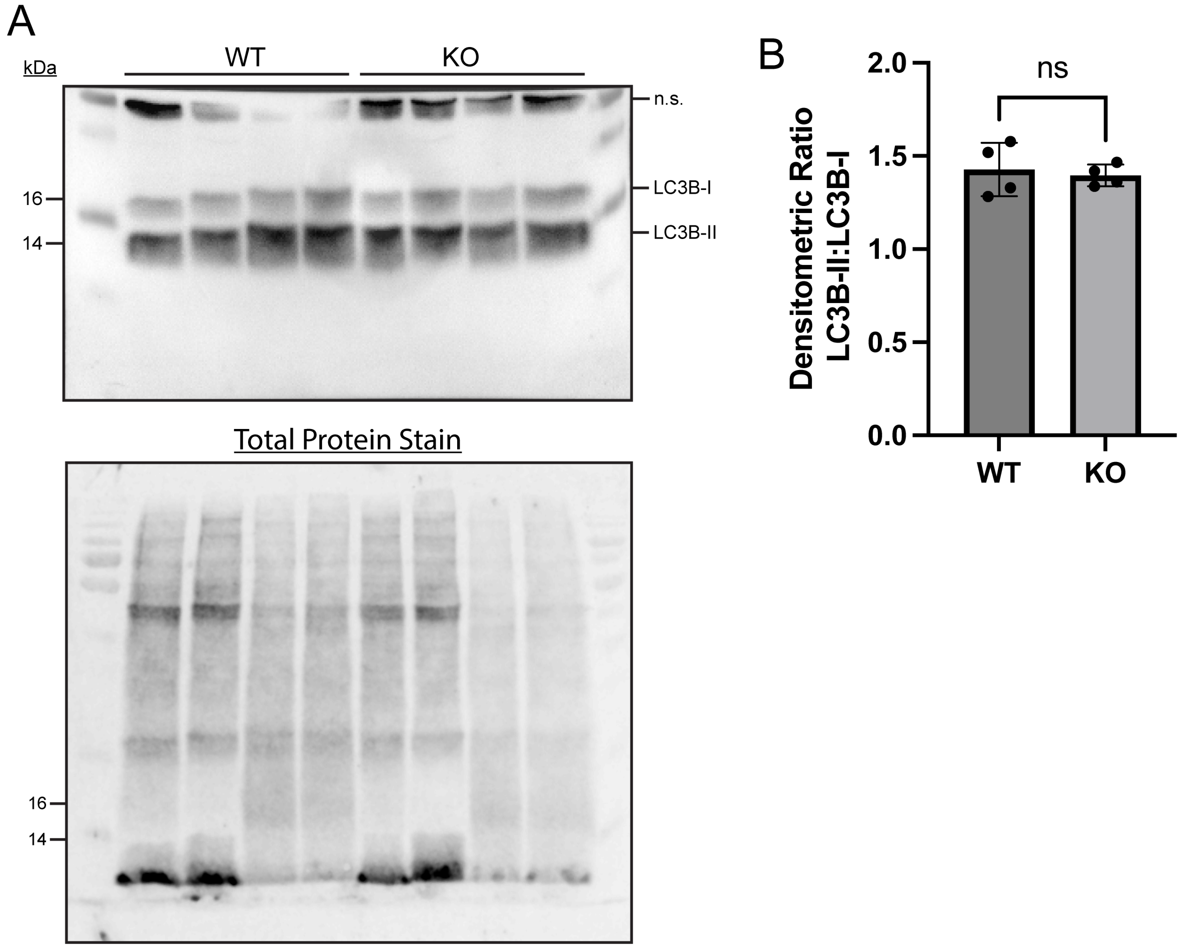

Supplementary Figure 3. Western blot shows no change in the LC3B-II:LC3B-I ratio in *mtmr5*-KO zebrafish. (A-B) Western blot analysis using whole-zebrafish lysates shows no change in the densitometric ratio between LC3B-II:LC3B-I in 7 days-post-fertilization (dpf) *mtmr5*-knockout (KO) compared to wild-type (WT) siblings. Each lane (A) or each dot (B) represents n=20 zebrafish (40 µg of total proteins), four lanes represent four independent experiments. Full blot images are presented, including the total protein stain. n.s. = non-specific. Densitometry was measured using Fiji ImageJ. Data are mean ± SEM. Unpaired two-tailed Student's t-test: *P<0.05; **P<0.01; ns, not significant.

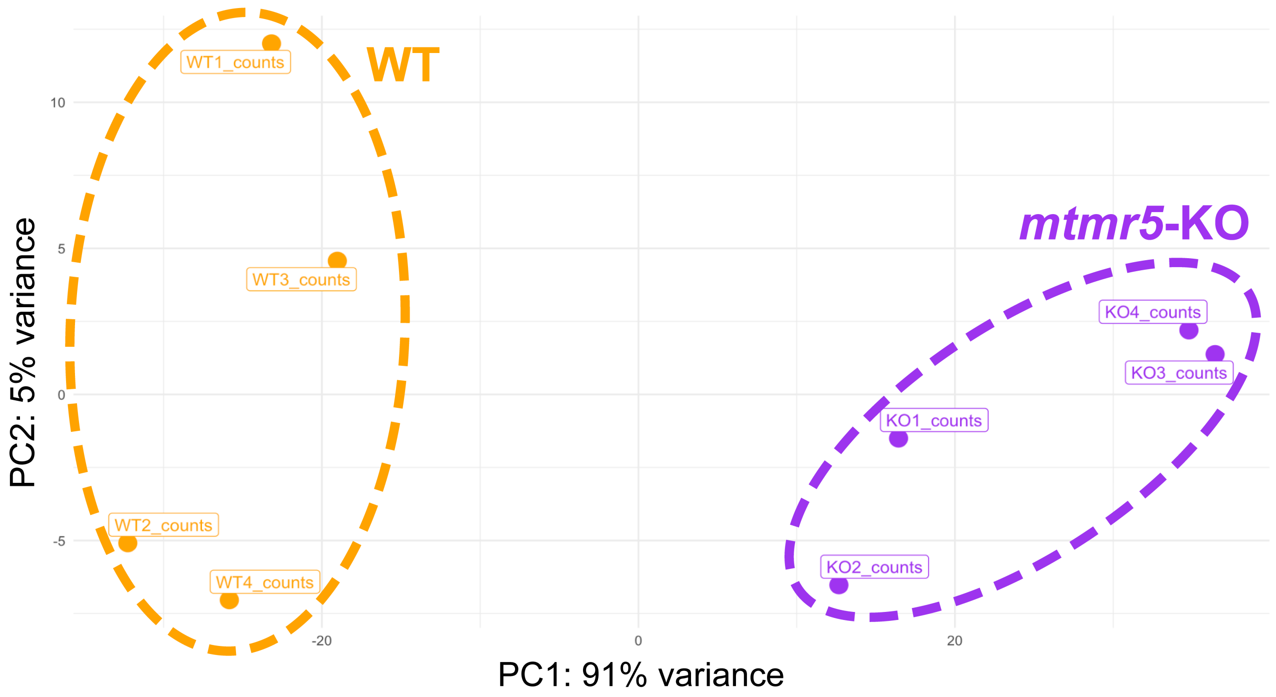

Supplementary Figure 4. Transcriptomic profile clustering of WT vs. *mtmr5*-KO bulk RNA-sequencing samples. Principal component analysis (PCA) of 7 days-post-fertilization (dpf) brain-enriched RNA-sequencing data from four wild-type (WT) and four *mtmr5*-knockout (KO) replicates. Each dot represents an individual RNA-sequencing sample replicate; WT = orange, KO = purple. Principal component 1 (PC1, x-axis) has an 91% variance, principal component 2 has a 5% variance (PC2, y-axis). The apparent WT (left) and KO (right) clusters represented by dashed lines indicates distinct transcriptomic profiles between the two conditions. Analysis performed using DESeq2 in R.

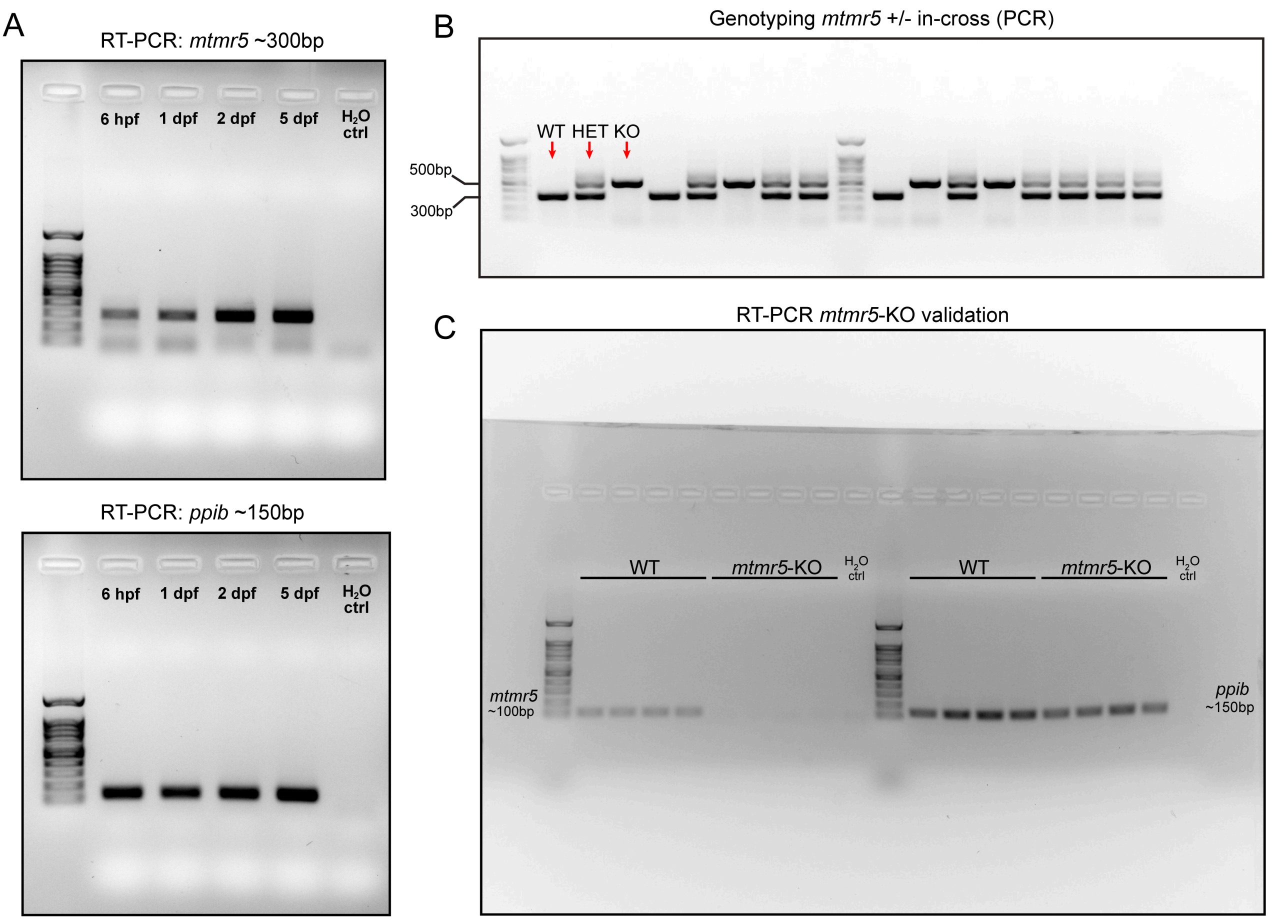

Supplementary Figure 5. Uncropped Gels from Figure 1 & Figure 2. (A) Full uncropped gels from Figure 1B, showing RT-PCR of WT cDNA using primers against *mtmr5* mRNA, *ppib* mRNA as a positive control, and H_20 (water) as a negative control. (B) Full uncropped gel from Figure 2C, showing genotyping of a *mtmr5*-heterozygous in-cross using a three-primer PCR method. (C) Full uncropped gel from Figure 2D, showing RT-PCR of WT and *mtmr5*-KO cDNA using primers against *mtmr5* mRNA, *ppib* mRNA as a positive control, and H_20 (water) as a negative control. WT = wild-type, HET = heterozygous, KO = knockout.
